# Mitochondria and Aging—The Role of Exercise as a Countermeasure

**DOI:** 10.3390/biology8020040

**Published:** 2019-05-11

**Authors:** Mats I Nilsson, Mark A Tarnopolsky

**Affiliations:** 1 Department of Pediatrics and Medicine, McMaster University Medical Center, Hamilton, ON L8S 4L8, Canada; mats.nilsson@exerkine.com or mnilsson7714@gmail.com; 2 Exerkine Corporation, McMaster University Medical Center, Hamilton, ON L8N 3Z5, Canada

**Keywords:** aging, exercise, mitochondria, aerobic, ROS, inflammation, senescence, lysosome, autophagy, mitophagy

## Abstract

Mitochondria orchestrate the life and death of most eukaryotic cells by virtue of their ability to supply adenosine triphosphate from aerobic respiration for growth, development, and maintenance of the ‘physiologic reserve’. Although their double-membrane structure and primary role as ‘powerhouses of the cell’ have essentially remained the same for ~2 billion years, they have evolved to regulate other cell functions that contribute to the aging process, such as reactive oxygen species generation, inflammation, senescence, and apoptosis. Biological aging is characterized by buildup of intracellular debris (e.g., oxidative damage, protein aggregates, and lipofuscin), which fuels a ‘vicious cycle’ of cell/DNA danger response activation (CDR and DDR, respectively), chronic inflammation (‘inflammaging’), and progressive cell deterioration. Therapeutic options that coordinately mitigate age-related declines in mitochondria and organelles involved in quality control, repair, and recycling are therefore highly desirable. Rejuvenation by exercise is a non-pharmacological approach that targets all the major hallmarks of aging and extends both health- and lifespan in modern humans.

## 1. Introduction

Mitochondria are the energy-producing organelles of nearly all eukaryotic cells, which arose ~1.5–2 billion years ago when a phototrophic α-proteobacterium was endocytosed by an ancestral eukaryote [1]. This endosymbiotic relationship is thought to have conferred significant evolutionary advantages to the anaerobic host at a time when Earth was becoming more oxygenated [2]. The increased energy availability allowed for expansion of the eukaryotic genome, enhanced protein expression, and more complex signaling pathways and cellular traits [3], allowing for the rise of complex life [4].

In terms of human evolution, the first marked increase in hominin brain size emerged ~2 million years ago concurrent with increased exploration (ranging, scavenging, and hunting), a dietary shift to higher quality/nutrient-dense food (meat), and technological sophistication [5,6]. Striding bipedalism, such as long-distance walking and running, is a unique human trait contingent upon aerobic prowess (e.g., lungs, heart, and muscles), and allowed for divergence from their apelike forbears to become successful hunters [7,8]. It is now widely accepted that the ability to deliver and utilize oxygen by the cardiorespiratory system and skeletal muscles, respectively (e.g., maximal aerobic capacity; VO_2_max), is a strong determinant of health and longevity in modern humans [9]. For example, runners have ~45–70% and ~30–50% reduced risk of mortality from cardiovascular disease (CVD) and cancer, respectively, and live 3–10% (2–8 years) longer than non-runners [10,11]. 

**Figure 1:**
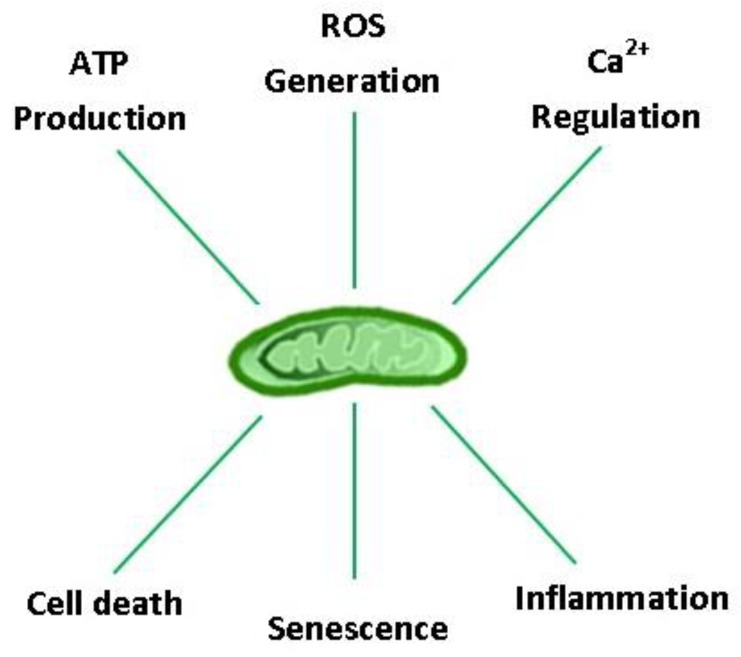
Major eukaryotic cell functions regulated by mitochondria. ATP: adenosine triphosphate; ROS: reactive oxygen species; Ca^2+^: calcium ion.

Considering the profound role of mitochondria in the evolution of aerobic life, it is not surprising that they hold a central position in cellular homeostasis and drive many aspects of the biological aging process. Aging is characterized by a progressive impairment of all body organs, including those that regulate VO_2_max and locomotion (e.g., cardiorespiratory, nervous, and musculoskeletal systems), resulting in a ~10% decline in aerobic capacity per decade in both males and females after ~ 30 years of age [12,13]. Post-mitotic cells are particularly susceptible to the ‘wear and tear’ of aging, as exemplified by the progressive build-up of intracellular debris over a lifetime [14]. Concomitant oxidative damage, protein aggregation, and lipofuscinogenesis are interrelated features of the aging process, neurodegenerative disease, and lysosomal storage disorders [14,15,16]. Collectively, these danger-associated molecular patterns (DAMPs) fuel a ‘vicious cycle’ of cell/DNA danger response activation (CDR and DDR, respectively), senescence, and systemic inflammation (‘inflammaging’) [17]. Organelles that regulate reactive oxygen species (ROS) production (mitochondria), protein quality control/repair (unfolded protein response: endoplasmic reticulum (UPR^ER^) and mitochondria (UPR^MT^)), and recycling (autophagosomes, lysosomes, and proteasomes) therefore constitute the cell’s major defense systems against aging. Arguably, no other organelle is more important than the mitochondria in this context because they provide the bulk of the energy needed to sustain the ‘physiologic reserve’ and regulate other vital functions for cell survival, including ROS production, inflammation, senescence, and apoptosis (Figure 1) [18,19,20]. Currently, physical activity (PA) and caloric restriction represent the only non-pharmacologic means to enhance health-span and life expectancy by their ability to coordinately rejuvenate the systems that drive the biological aging process [21,22]; however, exercise is the only factor confirmed to lower morbidity and all-cause mortality in epidemiological studies.

Herein, we highlight the integrative nature of cell aging, review the evidence for age-associated mitochondrial dysfunction, and discuss how habitual PA attenuates the biological aging process, specifically aerobic (AET) and resistance (RET) exercise training. Because mitochondria have been extensively studied in heart and muscle, the main organs that limit VO_2_max, we primarily focus on myocellular aging in this review.

## 2. Integrated Systems Hypothesis of Aging

Although over 300 hypotheses of aging have been proposed to date, with the vast majority focused on observable age effects (‘wear and tear’) or presumed root causes of age-associated pathology (‘primary damage’) [23], the common denominator across species has not yet been identified. The works of Schrödinger [24], Bortz [25], and Hayflick [26,27] collectively point to biological aging being a stochastic process occurring after reproductive maturity that is driven by entropy and results in progressive accumulation of random, irreparable losses in molecular fidelity. In line with the second law of thermodynamics, entropy is the tendency of a system to spontaneously disperse energy and evolve toward thermodynamic equilibrium, which is exemplified by a molecule’s altered energy state following breakage of its intra- and/or inter-molecular bonds. According to Hayflick [27], entropic changes are circumvented by the cell’s high-fidelity repair and replacement mechanisms until reproductive maturity, at which the rate of damage accumulation exceeds the rate of self-renewal. 

One of the most prominent hypotheses in the category of ‘primary damage’ is Denham Harman’s free radical theory of aging [28], originally conceptualized in 1954 but still garnering interest in the research community [29]. The original hypothesis posits that oxygen free radicals are the driving factors of aging, but has evolved to include all forms of reactive oxygen species (ROS) and mitochondria as the main source of ROS (oxidative stress and mitochondrial theories of aging, respectively) [28,30,31]. Subsequent observations of buildup of indigestible material in the lysosomes of post-mitotic cells (e.g., ferritin, mitochondrial fragments, and lipofuscin/‘age-pigment’) connected the aging process with an impairment in autolysosomal clearance (the mitochondrial–lysosomal axis theory of aging) [14]. Franceschi et al. further expanded on this integrated hypothesis by arguing that chronic, systemic inflammation at old age (inflammaging) is fueled by intracellular DAMPs originating from this ‘garbage catastrophe’ [32].

**Figure 2:**
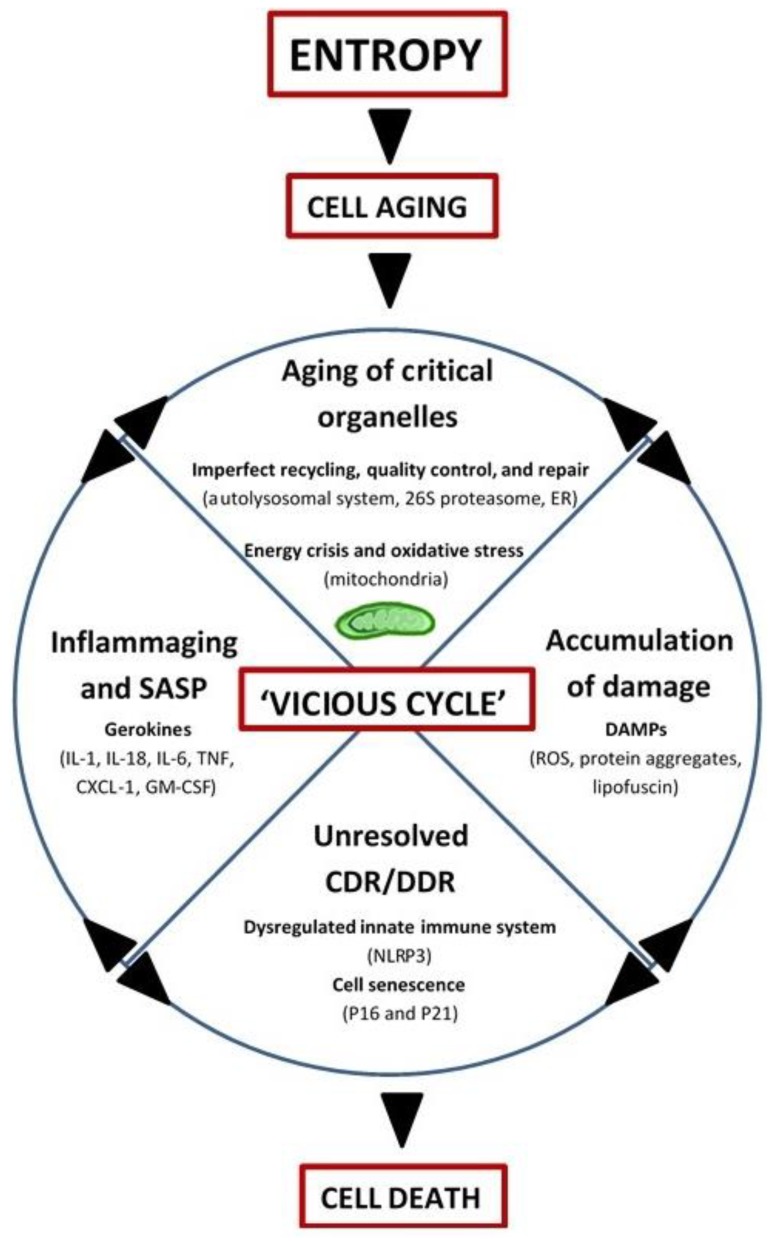
Integrated Systems Hypothesis of Aging (see text for details). DAMPs: danger-associated molecular patterns; ER: endoplasmic reticulum; CDR: cell danger response; DDR: DNA danger response; Inflammaging: chronic, low-grade inflammation with aging; SASP: senescence-associated secretory phenotype; Gerokines: cytokines, chemokines, growth factors, and proteases increased with aging; Vicious cycle: self-reinforcing feedback loop with detrimental outcome(s); NLRP3: inflammasome; P16: tumor suppressor protein P16^INK4A^/CDKN2A; P21: tumor suppressor protein P21^Cip1^/CDKN1A; IL: interleukin; TNF: tumor necrosis factor; CXCL-1: chemokine (C-X-C motif) ligand 1 (also KC and GROα); GM-CSF: granulocyte-macrophage colony-stimulating factor.

In summary, aging may be driven by entropy and manifests as a progressive accumulation of molecules with altered energy states, rendering them inactive or malfunctioning, prone to posttranslational modifications (e.g., oxidation, acetylation, methylation, glycation, etc.), cross-linking, and aggregation, and ultimately resistant to normal recycling mechanisms (for example, advanced glycation end products (AGEs) and lipofuscin). While mitochondria still remain central to the biological aging process in this view (by virtue of altered ROS and energy production), other organelles involved in recycling and quality control/repair also age, thus contributing to the ‘vicious cycle’ of debris accumulation, DAMP-activation of CDR/DDR, inflammation, and induction of cell-death (Figure 2).

## 3. Mitochondrial Respiration: Yin and Yang of Aerobic Life

From an aging perspective, thermodynamically unfavorable/endergonic processes, such as biosynthesis and repair, are essential to counteract inevitable energy dispersal by entropy. A consistent supply of energy in the form of adenosine triphosphate (ATP) is integral to maintain tissue order and function; a task that is governed by the mitochondria [33]. Mammalian mitochondria generate >80% of cellular ATP under normal conditions and are composed of ~1158 proteins encoded by the nuclear genome and to a lesser extent by mitochondrial DNA (mtDNA) (MitoCarta2.0 [34]). The 37 gene products transcribed by mtDNA (e.g., 2 ribosomal ribonucleic acids (rRNAs), 22 transfer ribonucleic acids (tRNAs), 13 protein sub-units (7 complex I, 1 complex III, 3 complex IV, and 2 complex V)) are synthesized within the organelle itself, while the vast majority of mitochondrial proteins are encoded by the nuclear genome, synthesized in the cytoplasm, and imported by the mitochondrial translocation machinery. Mitochondrial biogenesis, and by extension energy supply, cell homeostasis, and human longevity, rely on the synchronous and concerted action of these processes.

Aerobic energy production by mitochondria, referred to as oxidative phosphorylation (OXPHOS), consumes the vast majority of cellular oxygen and is driven by a series of redox reactions/electron transfers in the inner mitochondrial membrane (mitochondrial respiratory chain (MRC)) [35]. In this process, electrons are successively transferred from electron donors (reducing agents) generated by macronutrient oxidation (glucose, fatty acids, and amino acids) to successively more electronegative electron-acceptors (oxidizing agents) in order to establish a proton gradient to drive ATP synthesis. Ironically, molecular oxygen is not only essential for ATP synthesis, but also represents a major source of reactive oxygen species (ROS) in mammalian cells, making oxidative metabolism a double-edged sword requiring careful cellular coordination. 

As a natural by-product of respiration, ~0.2–2% of molecular oxygen undergoes a one-electron reduction into superoxide radicals in complexes I and III (O_2_^−•^), which may be further converted into membrane-permeable singlet oxygen (^1^ΔgO_2_ and ^2^∑gO_2_) or hydrogen peroxide (H_2_O_2_) [36,37,38,39]. Although mainly generated by complexes I and III, H_2_O_2_ and O_2_^−•^ are also produced by the monoamine oxidases and NADPH oxidases in mitochondria [39,40]. Transition metals in iron–sulfur clusters in the MRC and lysosomes may react with H_2_O_2_ to generate hydroxyl radicals (^•^OH; via Fenton-type reactions), which are short-lived but indiscriminate oxidants that are highly dangerous to biological organisms [20]. Superoxide may also become protonated into perhydroxyl radicals (HO_2_^•^) and have been proposed to play a central role in mediating the toxic side effects of aerobic respiration because of their high reactivity and membrane permeability [41]. Other potentially damaging molecules are also produced by the mitochondria, such as nitric oxide (NO^•^) and peroxynitrite (ONOO^−^), but are technically considered reactive nitrogen species (RNS).

Chronic overproduction of ROS can lead to oxidative damage, cell toxicity, and apoptosis, and is linked to neurodegenerative diseases, cancer, and aging [42,43]. Paradoxically, ROS are also integral for regulation of cell signaling pathways, gene expression, and exercise adaptations [20]. Consequently, the complete amelioration of pro-oxidants is not advantageous for cell viability or health [43,44]. ROS levels are thereby exquisitely fine-tuned by the cell’s principal enzymatic (EA) and non-enzymatic (NEA) antioxidant defense systems (EA: superoxide dismutases 1 and 2 (Cu/Zn-SOD and Mn-SOD, respectively), catalase, glutathione reductase, and glutathione peroxidases (GPx 1-4); NEA: reduced vs. oxidized glutathione (GSH: GSSG ratio), vitamin E, and vitamin C). Perturbations in the ‘redox state’ of the cell, generally defined as an imbalance between (pro-oxidants)/(anti-oxidants), predisposes towards oxidative damage [20]. Biological targets include lipids and proteins of cell membranes and nucleic acids of either genome being the most vulnerable in post-mitotic tissues. Oxidative modifications may lead to inactivation, fragmentation, and degradation of proteins, decomposition of membrane lipids, and significant RNA/DNA damage, including strand breaks, cross-links, and mutations, which predispose for senescence and cell-death [43].

## 4. Mitochondrial Aging

### 4.1. Oxidative Stress, mtDNA Mutagenesis, Apoptosis, and Respiration

Although direct evidence from human trials is lacking, mitochondrial O_2_^−•^ and H_2_O_2_ production increases with advancing age and is inversely correlated to lifespan in multiple mammalian species and flies [45,46,47,48]. Excessive mitochondrial ROS production (and/or reduced antioxidant capacity) is associated with oxidative damage, MRC dysfunction, loss of mitochondrial membrane potential (ΔΨ_m_), and induction of cell-death pathways in post-mitotic tissues of both prematurely (progeroid) and physiologically aged animal models. For example, mtDNA polymerase gamma-deficient mice (PolG; ↑ mtDNA mutagenesis) exhibit an accelerated aging phenotype (shorter lifespan, muscle atrophy, cardiomyopathy, anemia, thin dermis, gray fur, and kyphosis), deficits in OXPHOS function and ATP synthesis, and increased ROS-induced damage to mitochondrial proteins and nucleic acids [49]. Consistent with observations made in old Fisher 344 Brown Norway rats [48], reduced ΔΨ_m_ in PolG mice is associated with the release of pro-apoptotic factors and induction of apoptosis, which likely contributes to organ dysfunction and muscle wasting in this model [50,51,52]. As cogently summarized by others [53,54], ROS imbalance, Ca^2+^ dysregulation, and/or loss of ΔΨ_m_ may mediate mitochondrial outer membrane permeabilization and activation of intrinsic apoptotic pathways by opening of the mitochondrial permeability transition pore (mPTP) and the Bax/Bcl2-controlled mitochondrial apoptosis channel. ROS also contribute to telomere shortening and nuclear DNA instability (mainly in stem cells [55]), and genotoxic damage is a known activator of p53-mediated mPTP opening and apoptosis [56], which is the basis of the telomere-p53-mitochondrion model of aging [57]. In other words, several intrinsic (mitochondrial, ER, and lysosomal) and extrinsic (death receptor-induction by TNF-α and FasL) pathways may cooperate in myonuclear and satellite cell apoptosis, while mitochondria-driven cell death is believed to play the most important role in sarcopenia of aging [53,54,58,59]. 

Biological aging in humans is characterized by a progressive accumulation of oxidative damage and mutations to the mitochondrial genome from the third decade of life onward in several post-mitotic tissues (for example, muscle, heart, and brain) [31,60,61,62,63]. Concurrent with (or as a result of) increased ROS-induced damage and/or mtDNA mutagenesis, aging mitochondria display morphological abnormalities [30,64], lower MRC and OXPHOS activities [65,66], and impaired ATP synthesis [67,68]. Age-associated mitochondrial dysfunction, as assessed in vivo or at the whole tissue level [69], is attributable to intrinsic mitochondrial deficiency and a reduction in organellar number [68,70,71]. Due to the close proximity of mtDNA to the source of ROS, lack of protection by histones, and limited capacity for DNA repair [72,73], mtDNA is more susceptible to oxidative damage than nuclear DNA (nDNA), resulting in a nearly 20-fold higher mutation rate [74], including deletions [75,76,77,78,79,80], tandem duplications [81], and single base modifications [82]. In a series of landmark publications by the groups of Aiken and Turnbull, it was shown that clonal expansion of mtDNA mutations were linked to energy-deficient, cytochrome *c* oxidase-negative (COX^−^) areas within skeletal muscle that contained atrophied and broken myofibers with high apoptotic susceptibility [75,76,83,84,85,86,87]. In one elegant study, Bua et al. found that a significant number of *vastus lateralis* (VL) muscle fibers displayed a ‘ragged-blue phenotype’ (e.g., succinate dehydrogenase-hyperactive (SDH^++^) and COX^−^) in older humans (>90 years), and that >80% of the total mtDNA pool was mutated in affected fibers [76]. Other findings suggest that random deletions may be present in up to 70% of mtDNA molecules in VL muscle of ‘the oldest old’, primarily affecting MRC complexes that contain mtDNA-encoded subunits [88]. 

**Figure 3:**
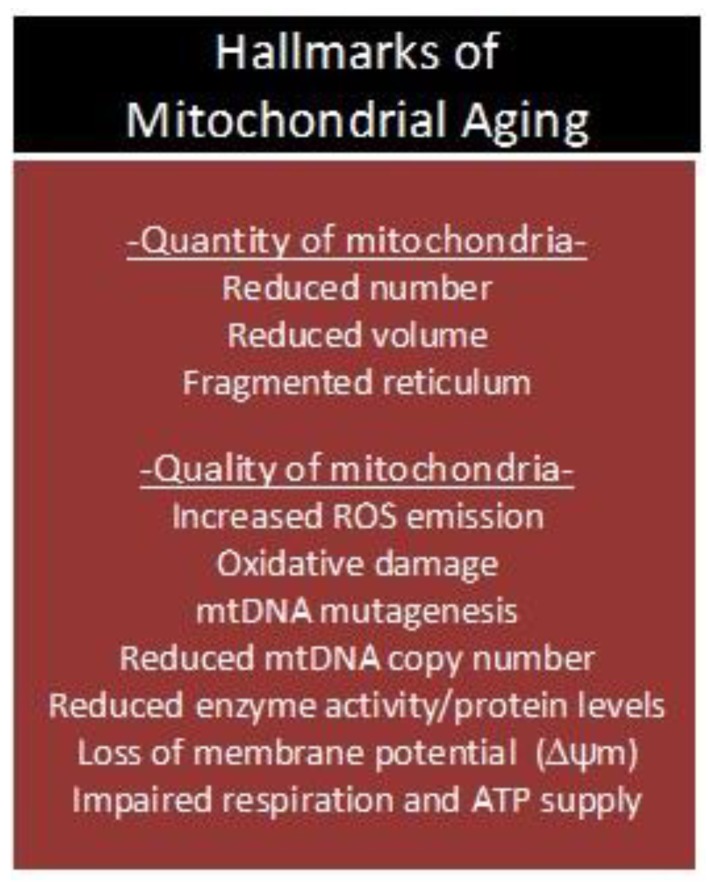
Age-associated deterioration in ‘quantity and quality’ of mitochondria. ROS: reactive oxygen species; mtDNA: mitochondrial DNA; ΔΨ_m_: mitochondrial membrane potential; ATP: adenosine triphosphate

Collectively, animal and human studies indicate that MRC dysfunction and OXPHOS deficits are common features of biological aging across multiple species (e.g., flies, mice, rats, dogs, monkeys, and humans) [89], and that a loss of ΔΨ_m_, redox imbalance, and mtDNA mutagenesis confer a significant challenge to a plethora of organ systems and cell functions in mammals (Figure 3). It is well-known that the major growth-regulatory processes in skeletal muscle (GRPs; synthesis, degradation, satellite cell function, and apoptosis) are sensitive to perturbations in ROS, Ca^2+^, ATP, and immunological homeostasis. Progressive dysfunction of organelles that regulate the aforementioned signaling molecules in skeletal muscle may therefore underlie the age-associated induction of intrinsic and extrinsic apoptotic pathways [53,54], reduction in proliferation and differentiation potentials of satellite cells [90], and desensitization to the anabolic and anti-proteolytic effects of insulin receptor (IR) stimulation [91,92,93,94]. Although these observations are consistent with the mitochondrial theory of aging [28,30,95], the question as to whether mitochondrial dysfunction drives the aging process and sarcopenia remains to be answered.

### 4.2. Garbage Catastrophe—The Role of Mitochondria

Recycling of biologic waste provides the cell with new building-blocks and substrates for energy metabolism; an integral housekeeping process predominately executed by the proteasome and lysosomes. Clearance of damaged organelles and macromolecules is critically important to maintain tissue homeostasis, particularly in post-mitotic cells that are unable to undergo waste dilution by cell division. Mitochondrial proteostasis is governed by an integrated network of pathways that include the organelles specialized in recycling and protein quality control (e.g., 26S proteasome, autolysosomal system, and PERK-mediated UPR^ER^) and mitochondria-specific QC mechanisms (fusion/fission, mitophagy, various proteases, and the GCN2-mediated UPR^MT^) [96,97]. Failure to maintain cellular clearance causes clumping of oxidatively damaged and misfolded proteins, formation of insoluble aggregates, and cell death by apoptosis or necrosis. The importance of efficient waste disposal is demonstrated by the fact that its disruption leads to neurodegenerative disease and lysosomal storage disorders; conditions linked to accelerated aging of neurons and muscle cells. Ablation of genes coding for lysosomal hydrolases or proteins that regulate intracellular waste delivery to lysosomes (e.g., autophagy) is associated with autophagic blockage, mitochondrial dysfunction, and tissue deterioration. In the case of acid α-glucosidase deficiency (Pompe disease), failure to clear lysosomal glycogen leads to cardiorespiratory insufficiency, muscle wasting, and premature death [98]. Conversely, pharmacological or genetic manipulations that prolong lifespan in model organisms typically activate cellular clearance pathways, and their inhibition may negate the life-extending effects, as in the case of caloric restriction [99].

A unifying feature in the pathogenesis of mammalian aging and accelerated aging conditions is the progressive deposition of cytotoxic debris impervious to lysosomal and proteasomal degradation. Age-related functional declines in autophagy, including macroautophagy and microautophagy (and likely aggrephagy), are linked to impaired mitochondrial turnover, protein aggregation, and accumulation of lipofuscin [14,16,99,100,101,102,103]. Lipofuscin, or ‘aging pigment’, is a degradation-resistant, redox-active biomolecule composed of oxidized proteins (30–70%), lipids (20–50%), and transition metals (iron, copper etc.) and increases with advancing age in lysosomes of post-mitotic cells [100,104,105,106]. In humans, lipofuscin has been demonstrated in heart, liver, kidney, and skin, but is believed to play the most fundamental role in the aging process of neurons and muscle cells [100]. Motor neurons in the anterior horn of the spinal cord, which innervate muscles necessary for voluntary movement of the limbs and trunk, appear particularly susceptible to lipofuscin deposition [104]. In addition, lipofuscin in skeletal muscle has been proposed to be a more robust marker of age-induced pathology compared to oxidative stress/damage [107]. 

Due to degradation of iron-containing macromolecules (ferritin, myoglobin, cytochrome *c* (e.g., mitochondrial complexes)), lysosomes, and by extension lipofuscin, contain significant amounts of low-mass, reactive iron (Fe^2+^), which catalyze Fenton-type reactions (H_2_O_2_ → ^•^OH)[108]. Hydroxyl radicals are extremely harmful and induce ubiquitous damage to biologic material, including peroxidation and permeabilization of the lysosomal membrane (LMP). Aspartic and cysteine cathepsins are released from LMP and cleave targets in the apoptotic pathway (Bid, Bcl-2 family members, caspase 8, and XIAP), culminating in activation of apoptosis, amplification of the apoptotic response, and/or necrosis [109]. Other known LMP inducers are DNA damage, lysosomotropic agents, calpain 1, and extrinsic stimuli such as death receptor ligands and signaling enhancers (TNF-α, FasL, IFN-γ) [109]. In addition to being independent activators of apoptosis, significant crosstalk occurs between lysosomes, mitochondria, and the ER in response to cellular stress via H_2_O_2_, cathepsins, and Ca^2+^. According to the mitochondrial–lysosomal axis theory of aging, mitochondrial ROS serves as an accelerant of lipofuscinogenesis, which impairs lysosomal degradative capacity and recycling of damaged mitochondria, further perpetuating redox imbalance, cytotoxicity, and debris aggregation [14]. Collectively, mitochondria and lysosomes generate the vast majority of ‘accelerating agents’ for oxidation, aggregation, and lipofuscinogenesis (ROS and Fe^2+^, respectively), play major roles in the induction of cell death, and likely contribute significantly to sarcopenia and the biological aging process.

### 4.3. Inflammaging—The Role of Mitochondria

Inflammation is a basic biological response to prevent, limit, and repair damage by invading pathogens or endogenous biomolecules. Cell stress and infectious agents trigger transmembrane (Toll-like (TLR) and C-type lectin) and cytosolic (NOD-like (NLR), RIG-I-like (RLR), and PYHIN protein family) signaling receptors in immune and non-immune cells, which activate intracellular and humoral components of the innate and acquired immune systems [110]. While the transient inflammatory response is beneficial (removal of pathogens, mitigation of injury, and clearance of dying cells), persistent inflammation is associated with tissue dysfunction and pathology (obesity, type 2 diabetes, atherosclerosis, asthma, and neurodegenerative diseases) [111]. Chronic low-grade inflammation (inflammaging) is a hallmark of biological aging and is characterized by a 2 to 4-fold increase in circulating cytokines, chemokines, growth factors, and proteases, collectively termed ‘gerokines’, which may be broadly classified into pro- (TNF-α, IL-1α/β, IL-8, IFNγ, VEGF, etc.) and anti-inflammatory (IL-2, IL-4, IL-10, IL-13, TGF-β, etc.) factors [17,32]. Inflammaging is attributed to DAMP-activation of the innate immune response, cell senescence (e.g., SASP; senescence-associated secretory phenotype), and immunosenescence, and has been linked to an elevation in all-cause mortality and sarcopenia [112,113,114,115,116,117,118]. 

In 2002, Jürg Tschopp discovered a molecular platform that mediates the induction of the innate immune response in myeloid (monocytes, macrophages, dendritic cells, and neutrophils) and nonmyeloid cells (nerve, muscle, heart, endothelial etc.) [119,120]. The multi-protein complex, referred to as the inflammasome, is a cytosolic receptor that senses pathogen- and damage-associated molecular patterns (PAMPs and DAMPs, respectively), activates caspase-1, and causes IL-1β/IL-18 maturation. Subsequent secretion of IL-1β/IL-18 recruits immune cells to the site of damage, which leads to further release of cytokines and chemokines (TNF- α, IL-1β etc.), cell death, and phagocytosis of apoptotic bodies. NLRP3, the most widely studied inflammasome, requires a priming step by NF-κB and a danger/pathogen signal to become fully activated [121]. Tschopp’s group demonstrated that ROS overgeneration by mitochondria, induced by inhibition of mitophagy (via 3-MA and Beclin1/ATG5 knockdown) or complex I and III inactivation (via rotenone and anti-mycin, respectively), activated the NLRP3 inflammasome and promoted IL-1β secretion [122,123]. Other DAMPs, including mitochondrial- (mtDNA, cardiolipin, mitofusins, and the mitochondrial antiviral-signaling protein (MAVS)), lysosomal- (cathepsins), and ER-derived (Ca^2+^), directly regulate NLRP3 activity or are integral in the recruitment and docking of the inflammasome to the mitochondria [110,124]. Oxidized mtDNA released during apoptosis is a known inducer of NLRP3 [125], increases gradually after the fifth decade of life, and is positively correlated to systemic proinflammatory cytokine levels [115]. Failure to recycle damaged mitochondria causes ROS overgeneration, mtDNA damage, and exacerbation of the inflammatory response (as shown by Tschopp et al.). Taken together, these data suggest that age-associated danger signals generated from mitochondria, lysosomes, and the ER contribute to inflammaging and sarcopenia [113].

## 5. The Anti-Aging Benefits of Physical Activity

In light of the hormetic effects of low levels and/or pulses of oxidative stress [20,126], aging intervention strategies should be aimed at dampening (but not ameliorating) persistent ROS overgeneration and removing oxidative damage and protein aggregates as expediently as possible, which would limit the formation of waste products refractory to normal enzyme catalysis and inflammation. Attenuation of the major hallmarks of aging will not halt entropy per se, but will delay downstream pathology, extend health-span, and add longevity. Accrual of an excess physiologic reserve before the reproductive peak in humans (20–30 years), and maintenance of this reserve capacity by efficient repair and recycling in adulthood (40 years onwards), are synonymous with life extension, but may necessitate a combined approach of pharmacotherapy, rejuvenative biotechnology, and lifestyle modification. The feasibility of using non-exercise strategies for life extension has been discussed elsewhere [127]. 

### 5.1. Acute Exercise is Hormesis

Acute contractile activity is a hormetic stress stimulus that temporarily alters intracellular danger signals (ROS, Ca^2+^, pH, and hypoxia), lowers cellular energy state (NAD^+^/NADH and AMP/ATP), and promotes release of hormones and circulatory factors (‘exerkines’), which synchronously activate signaling pathways that stimulate mitochondrial biogenesis (CaMK II, PGC-1α, SIRT1, and AMPK), antioxidant defense (Nrf2-Keap1, NF-κB, and MAPK), waste recycling (autophagy (ULK1-Beclin1) and 26S proteasome (FOXO3a)), and the immune response (IL-1β, IL-18, IL-6, IL-10, IL-1ra, sTNF-R, etc.) [20,21,128,129,130,131,132,133,134,135,136,137,138,139]. Anabolic GRPs, mainly mediated by Akt-mTOR signaling (e.g., protein synthesis), are activated following exercise concomitant with energy repletion, and may stay elevated for 1–2 days in older adults [140]. Consistent with the concept of hormesis [44,141], repeated exposure to a single-stress stimulus such as exercise improves stress resistance and immunity, rejuvenates mitochondria (increased biogenesis, recycling, and damage removal), and increases the organ functional reserve [17]. 

### 5.2. Mitochondrial Rejuvenation

The long-term benefits of PA are multi-systemic (muscular, nervous, vascular, endocrine, and immune systems) and culminate in reduced all-cause mortality and enhanced longevity (e.g., ~3–10% in average life expectancy) [10,11]. AET is considered the gold standard to improve mitochondrial biogenesis, insulin sensitivity, and cardiorespiratory fitness across all age groups. In older adults, AET partially reverses mitochondrial dysfunction by augmenting mtDNA copy number, mitochondrial transcript and protein expression, oxidative enzyme function, ATP synthesis, and total mitochondrial volume [142,143,144]. Short et al. demonstrated that the capacity for mitochondrial biogenesis (e.g., PGC-1α, NRF1, and TFAM), mitochondrial gene expression (COX IV and ND4), and Kreb’s cycle/MRC enzyme activities (CS and COX) may be enhanced by AET regardless of age [143]. Indeed, 12 weeks of progressive moderate-intensity AET (50–70% VO2 max) increased total mitochondrial content (mtDNA and cardiolipin), MRC function (NADH oxidase and succinate oxidase), and HOMA-IR (Homeostatic Model Assessment of Insulin Resistance) in older adults [142]. In the latter study, both pools of mitochondria (e.g., subsarcolemmal and intermyofibrillar) were responsive to contractile activity and the majority of mitochondrial variables were improved by >50%. Although findings by Broskey et al. suggest that AET-induced mitochondrial benefits are largely ascribed to higher mitochondrial volume density [144], other studies (both cross-sectional and longitudinal) indicate that the intrinsic quality of individual mitochondria may also be enhanced by AET [145,146,147].

RET is generally considered to have minimal effects on mitochondrial biogenesis, but our group and others have clearly shown that strength training rejuvenates the mitochondrial transcriptome profile, enhances MRC and antioxidant enzyme activities, and reduces oxidative damage in skeletal muscle of older adults [148,149,150,151,152,153]. A study by Jubrias et al. demonstrated that gains in mitochondrial volume density may even be greater following RET vs. AET in elderly (30% vs. 10%, respectively) [146], and one report suggests that mitochondrial adaptations are similar regardless of exercise mode [154]. Porter and Rasmussen found that the intrinsic quality of mitochondria is improved by regular strength training, with a potential shift in the relative contribution of complex I and complex II to maximal electron transfer [155]. Data from our studies suggest that RET-induced mitochondrial benefits are partially mediated by activation of satellite cells, which fuse with the mature myofiber and bring in wildtype mtDNA to ‘dilute down’ the mutant mtDNA pool [153]. The concept of mtDNA shifting following muscle overload and subtle myofiber injury by concentric and eccentric contractile activity, respectively, was first introduced by Taivassalo et al. and tested in mitochondrial disease patients [156,157]. We have now expanded this concept and demonstrated that progressive RET (50–75% of one-repetition maximum (1-RM)) lowers mtDNA deletions and increases lean mass, muscle strength, and function in older adults [152,153].

### 5.3. Intracellular Garbage Clearance

Aging is associated with ROS overgeneration that overwhelms antioxidant defense systems and leads to oxidative modifications of proteins, lipids, and nDNA/mtDNA [158]. Oxidative damage and other DAMPs fuel a vicious cycle that culminates in blunted recycling, debris accumulation, and inflammation. Oxidative stress reduction is thereby a key aspect of anti-aging therapies and a substantial amount data supports the antioxidant role of PA [20,158]. Regular training induces a shift from fast to intermediate muscle fiber types (e.g., glycolytic → more oxidative), which strengthens antioxidant defense and protects against buildup of damage [20]. Importantly, these observations are not limited to the muscles, as PA has been shown to have multi-systemic benefits on diverse tissues (skin and brain, for example), including reductions in nDNA/mtDNA adducts, AGE cross-links, and amyloid plaques [159,160,161]. 

Although the effects of contractile activity on quality control, repair, and recycling mechanisms remain largely unknown, several research teams have contributed to an increased understanding of how exercise modulates autolysosomal and 26S proteasomal pathways. Collectively, animal and human studies suggest that aerobic exercise reduces the cellular energy state, which stimulates lysosomal biogenesis, macroautophagy, proteasomal activity, and mitochondrial recycling via activation of TFEB (CLEAR network), ULK1-Beclin1, and FOXO transcription factors [132,162,163,164]. The magnitude of the response appears to be dependent upon the duration of exercise and nutrient status (e.g., fed or fasted state) [132]. In a study by Pagano et al, it was shown that aerobic exercise sequentially activates AMPK (preceding Akt-mTOR inhibition), ULK1, and FOXO3, leading to an increased LC3BII/LC3BI ratio, expression of E3 ubiquitin ligases (MuRF1 and MAFbx), and enhanced mitochondrial turnover (Mul1 and DRP1)[131]. Additionally, the integral role of Bcl2-mediated activation of Beclin1 for exercise-induced autophagy has previously been demonstrated in multiple tissues by Beth Levine’s group (muscle, heart, liver, pancreas, adipose tissue, and brain) [129,130]. Importantly, basal autophagic flux is improved in skeletal muscle following AET, concomitant with a shift from glycolytic to oxidative myofiber phenotype [165]. Data from our laboratory suggest that AET also augments autophagic debris removal in LSDs such as Pompe disease [15]. Conversely, RET may preferentially modulate the ubiquitin-proteasome pathway [132], but more research is needed to determine the effects of exercise mode and training intensity on recycling/repair processes.

### 5.4. Boosting the Immune System

High-intensity and unaccustomed exercise may cause tissue damage, elevated levels of pro- and anti-inflammatory factors, and delayed onset muscle soreness (DOMS) [17,166,167]. Typically, the acute inflammatory response is followed by a healing phase, structural remodeling, and muscle adaptation, which mitigates DOMS from subsequent exercise sessions. Over time, exercise leads to physiological adaptations into a more stress-resistant, homeostatic level, which protects against age-related chronic diseases such as systemic inflammation and cancer [17].

Although the anti-inflammatory benefits of PA are traditionally attributed to a reduction in visceral fat mass and/or induction of an anti-inflammatory environment with each bout of exercise [137,139], it is plausible that mitochondrial rejuvenation in multiple cell populations concomitantly enhances immunity via enhanced control of the inflammasome (e.g., ↓ DAMP-mediated activation). Considering that skeletal muscle is an endocrine organ that makes up ~35–50% of total body mass, immune benefits in older adults may be partially mediated by preservation of muscle mass. Results from randomized controlled trials indicate that AET and combined AET/RET boost the vaccination response, reduce circulatory levels of pro-inflammatory cytokines, and augment proliferative capacity and/or function of multiple cell types in the innate and adaptive immune systems [118]. Our group recently demonstrated that lifelong AET potently dampens inflammaging, including master regulators of cytokine cascade and tumorigenesis (IL1-α/β, TNF-α, and IL-6), which partially preserved muscle mass, protected against multi-systemic cancers, and enhanced health-span of naturally-aged mice [17]. Lastly, the anti-inflammatory effects of strength training in isolation are understudied, but there is sufficient evidence of improved immune function following long-term RET in frail elderly [168,169].

## 6. Conclusions

Concomitant oxidative damage, protein aggregation, lipofuscinogenesis, and inflammation are unifying features of the normal aging process, neurodegenerative disease, and lysosomal storage disorders. Activation of clearance pathways extends lifespan in multiple species, collectively suggesting that the ability to neutralize cytotoxins, recycle debris, and repair stress-induced damage is integral for survival. Although the ‘ground zero’ of aging may be entropy, we propose that the rate of aging is predominately dictated by the organelles/processes that govern the most critical needs of the cell, such as energy production (mitochondria), recycling (autophagosome, lysosome, and 26S proteasome), and quality control (UPR^ER^ and UPT^MT^). Given their importance in eukaryotic evolution, cell homeostasis, and growth, mitochondria may be considered the ‘hubs of aerobic life’, and are therefore assigned a central role in the Integrated Systems Hypothesis of Aging.

An impressive body of knowledge over the last 50 years unequivocally proves that regular exercise lowers all-cause and cardiovascular mortality risks, enhances health and longevity, and that an inactive lifestyle is inherently unsafe. Both major types of exercise, aerobic and resistance training, bestow multi-systemic benefits and protect against the major hallmarks of aging, including mitochondrial dysfunction, recycling deficiency, impaired quality control, and systemic inflammation, thus providing a compelling argument in support of exercise as a front-line modality to decelerate the aging process.

## References

[1] Kurland C.G., Andersson S.G.E. (2000). Origin and Evolution of the Mitochondrial Proteome. Microbiol. Mol. Biol. Rev..

[2] Holland H. (2006). The oxygenation of atmosphere and oceans. Philos. Trans. R. Soc. Lond. B Biol. Sci..

[3] Lane N. (2014). Bioenergetic Constraints on the Evolution of Complex Life. Cold Harb. Perspect. Boil..

[4] Stamati K., Mudera V., Cheema U. (2011). Evolution of oxygen utilization in multicellular organisms and implications for cell signalling in tissue engineering. J. Tissue Eng..

[5] Burini R.C., Leonard W.R. (2018). The evolutionary roles of nutrition selection and dietary quality in the human brain size and encephalization. Nutrire.

[6] Wood B., Collard M. (1999). The Human Genus. Science.

[7] Bramble D.M., Lieberman D.E. (2004). Endurance running and the evolution of Homo. Nature.

[8] Pontzer H. (2017). Economy and Endurance in Human Evolution. Curr. Biol..

[9] Kodama S., Saito K., Tanaka S., Maki M., Yachi Y., Asumi M., Sugawara A., Totsuka K., Shimano H., Ohashi Y. (2009). Cardiorepiratory fitness as a quantitative predictor of all-cause mortality and cardiovascular events in healthy men and women: A meta-analysis. JAMA.

[10] Reimers C.D., Knapp G., Reimers A.K. (2012). Does physical activity increase life expectancy? A review of the literature. J. Aging Res..

[11] Lee D., Brellenthin A., Thompson P., Sui X., Lee I., Lavie C. (2017). Running as a key lifestyle medicine for longevity. Prog. Cardiovasc. Dis..

[12] Pimentel A.E., Gentile C.L., Tanaka H., Seals D.R., Gates P.E. (2003). Greater rate of decline in maximal aerobic capacity with age in endurance-trained than in sedentary men. J. Appl. Physiol..

[13] Tanaka H., Desouza C.A., Jones P.P., Stevenson E.T., Davy K.P., Seals D.R. (1997). Greater rate of decline in maximal aerobic capacity with age in physically active vs. sedentary healthy women. J. Appl. Physiol..

[14] Terman A., Kurz T., Navratil M., Arriaga E.A., Brunk U.T. (2010). Mitochondrial turnover and aging of long-lived postmitotic cells: The Mitochondrial-Lysosomal Axis Theory of Aging. Antioxid. Redox Signal..

[15] Nilsson M., MacNeil L., Kitaoka Y., Suri R., Young S., Kaczor J., Nates N., Ansari M., Wong T., Ahktar M. (2015). Combined aerobic exercise and enzyme replacement therapy rejuvenates the mitochondrial-lysosomal axis and alleviates autophagic blockage in Pompe disease. Free Radic. Biol. Med..

[16] Hyttinena J.M., Amadiob M., Viiri J., Pascaleb A., Salminenc A., Kaarnirantaa K. (2014). Clearance of misfolded and aggregated proteins by aggrephagy and implications for aggregation diseases. Ageing Res. Rev..

[17] Nilsson M.I., Bourgeois J.M., Nederveen J.P., Leite M.R., Hettinga B.P., Bujak A.L., May L., Lin E., Crozier M., Rusiecki D.R. (2019). Lifelong aerobic exercise protects against inflammaging and cancer. PLoS ONE.

[18] Mehta M.M., Weinberg S.E., Chandel N.S. (2017). Mitochondrial control of immunity: Beyond ATP. Nat. Rev. Immunol..

[19] Marzetti E., Calvani R., Cesari M., Buford T.W., Lorenzi M., Behnke B.J., Leeuwenburgh C. (2013). Mitochondrial dysfunction and sarcopenia of aging: From signaling pathways to clinical trials. Int. J. Biochem. Cell Biol..

[20] Powers S.K., Jackson M.J. (2008). Exercise-induced oxidative stress: Cellular mechanisms and impact on muscle force production. Physiol. Rev..

[21] Garatachea N., Pareja-Galeano H., Sanchis-Gomar F., Santos-Lozano A., Fiuza-Luces C., Moran M., Emanuele E., Joyner M.J., Lucia A. (2014). Exercise attenuates the major hallmarks of aging. Rejuvenation Res..

[22] Fiuza-Luces C., Garatachea N., Berger N., Lucia A. (2013). Exercise is the real polypill. Physiology.

[23] Medvedev Z.A. (1990). An attempt at a rational classification of theories of ageing. Biol. Rev..

[24] Schrodinger E. (1947). What is life. The Physical Aspect of the Living Cell.

[25] Bortz W.M. (1986). Aging as entropy. Exp. Gerontol..

[26] Hayflick L. (2004). Aging: The reality: Anti-aging is an oxymoron. J. Gerontol. Ser. A Biol. Sci. Med Sci..

[27] Hayflick L. (2007). Entropy explains aging, genetic determinism explains longevity, and undefined terminology explains misunderstanding both. PLoS Genet..

[28] Harman D. (1972). The biologic clock: The mitochondria. J. Am. Geriatr. Soc..

[29] Sanz A., Pamplona R., Barja G. (2006). Is the mitochondrial free radical theory of aging intact?. Antioxid. Redox Signal..

[30] Miquel J., Economos A.C., Fleming J., Johnson J.E.J. (1980). Mitochondrial role in cell aging. Exp. Gerontol..

[31] Bokov A., Chaudhuri A., Richardson A. (2004). The role of oxidative damage and stress in aging. Mech. Ageing Dev..

[32] Franceschi C., Garagnani P., Vitale G., Capri M., Salvioli S. (2017). Inflammaging and ‘Garb-Aging’. Trends Endocrinol. Metab..

[33] Gray M. (1993). Origin and evolution of organelle genomes. Curr. Opin. Genet. Dev..

[34] Calvo S., Clauser K., Mootha V. (2016). MitoCarta2.0: An updated inventory of mammalian mitochondrial proteins. Nucleic Acids Res..

[35] Rolfe D.F., Brown G.C. (1997). Cellular energy utilization and molecular origin of standard metabolic rate in mammals. Physiol. Rev..

[36] Harper M., Bevilacqua L., Hagopian K., Weindruch R., Ramsey J. (2004). Ageing, oxidative stress, and mitochondrial uncoupling. Acta Physiol. Scand..

[37] Muller F.L., Liu Y., Van Remmen H. (2004). Complex III releases superoxide to both sides of the inner mitochondrial membrane. J. Biol. Chem..

[38] Chance B., Sies H., Boveris A. (1979). Hydroperoxide metabolism in mammalian organs. Physiol. Rev..

[39] Wong H.-S., Benoit B., Brand M.D. (2019). Mitochondrial and cytosolic sources of hydrogen peroxide in resting C2C12 myoblasts. Free Radic. Biol. Med..

[40] Maggiorani D., Manzella N., Edmondson D.E., Mattevi A., Parini A., Binda C., Mialet-Perez J. (2017). Monoamine Oxidases, Oxidative Stress, and Altered Mitochondrial Dynamics in Cardiac Ageing. Oxidative Med. Cell. Longev..

[41] De Grey A.D. (2002). HO_2_•: The Forgotten Radical. DNA Cell Biol..

[42] Mattson M.P. (2008). Hormesis defined. Ageing Res. Rev..

[43] Kohen R., Nyska A. (2002). Invited review: Oxidation of biological systems: Oxidative stress phenomena, antioxidants, redox reactions, and methods for their quantification. Toxicol. Pathol..

[44] Ristow M., Zarse K. (2010). How increased oxidative stress promotes longevity and metabolic health: The concept of mitochondrial hormesis (mitohormesis). Exp. Gerontol..

[45] Sohal R., Ku H., Agarwal S., Forster M., Lal H. (1994). Oxidative damage, mitochondrial oxidant generation, and antioxidant defenses during aging and in response to food restriction in the mouse. Mech. Ageing Dev..

[46] Sohal R., Sohal B., Orr W. (1995). Mitochondrial superoxide and hydrogen peroxide generation, protein oxidative damage, and longevity in different species of flies. Free Radic. Biol. Med..

[47] Ku H., Brunk U., Sohal R. (1993). Relationship between mitochondrial superoxide and hydrogen peroxide production and longevity of mammalian species. Free Radic. Biol. Med..

[48] Chabi B., Ljubicic V., Menzies K.J., Huang J.H., Saleem A., Hood D.A. (2008). Mitochondrial function and apoptotic susceptibility in aging skeletal muscle. Aging Cell.

[49] Dai D.F., Chen T., Wanagat J., Laflamme M., Marcinek D.J., Emond M.J., Ngo C.P., Prolla T.A., Rabinovitch P.S. (2010). Age-dependent cardiomyopathy in mitochondrial mutator mice is attenuated by overexpression of catalase targeted to mitochondria. Aging Cell.

[50] Hiona A., Sanz A., Kujoth G.C., Pamplona R., Seo A.Y., Hofer T., Someya S., Miyakawa T., Nakayama C., Samhan-Arias A.K. (2010). Mitochondrial DNA mutations induce mitochondrial dysfunction, apoptosis and sarcopenia in skeletal muscle of mitochondrial DNA mutator mice. PLoS ONE.

[51] Kujoth G.C., Hiona A., Pugh T.D., Someya S., Panzer K., Wohlgemuth S.E., Hofer T., Seo A.Y., Sullivan R., Jobling W.A. (2005). Mitochondrial DNA mutations, oxidative stress, and apoptosis in mammalian aging. Science.

[52] Trifunovic A., Hansson A., Wredenberg A., Rovio A.T., Dufour E., Khvorostov I., Spelbrink J.N., Wibom R., Jacobs H.T., Larsson N.G. (2005). Somatic mtDNA mutations cause aging phenotypes without affecting reactive oxygen species production. Proc. Natl. Acad. Sci. USA.

[53] Marzetti E., Privitera G., Simili V., Wohlgemuth S.E., Aulisa L., Pahor M., Leeuwenburgh C. (2010). Multiple Pathways to the Same End: Mechanisms of Myonuclear Apoptosis in Sarcopenia of Aging. Sci. J..

[54] Alway S.E., Siu P.M. (2008). Nuclear apoptosis contributes to sarcopenia. Exerc. Sport Sci. Rev..

[55] Von Zglinicki T. (2002). Oxidative stress shortens telomeres. Trends Biochem. Sci..

[56] Mihara M., Erster S., Zaika A., Petrenko O., Chittenden T., Pancoska P., Moll U.M. (2003). p53 has a direct apoptogenic role at the mitochondria. Mol. Cell.

[57] Sahin E., DePinho R.A. (2013). Axis of ageing: Telomeres, p53 and mitochondria. Nat. Rev. Mol. Cell Biol..

[58] Boya P., Kroemer G. (2008). Lysosomal membrane permeabilization in cell death. Oncogene.

[59] Szegezdi E., Logue S.E., Gorman A.M., Samali A. (2006). Mediators of endoplasmic reticulum stress-induced apoptosis. EMBO Rep..

[60] Hayakawa M., Hattori K., Sugiyama S., Ozawa T. (1992). Age-associated oxygen damage and mutations in mitochondrial DNA in human hearts. Biochem. Biophys. Res. Commun..

[61] Hayakawa M., Torii K., Sugiyama S., Tanaka M., Ozawa T. (1991). Age-associated accumulation of 8-hydroxydeoxyguanosine in mitochondrial DNA of human diaphragm. Biochem. Biophys. Res. Commun..

[62] Mecocci P., Fan G., Fulle S., MacGarvey U., Shinobu L., Polidori M.C., Cherubini A., Vecchiet J., Senin U., Beal M.F. (1999). Age-dependent increases in oxidative damage to DNA, lipids, and proteins in human skeletal muscle. Free Radic. Biol. Med..

[63] Parise G., Kaczor J.J., Mahoney D.J., Phillips S.M., Tarnopolsky M.A. (2004). Oxidative stress and the mitochondrial theory of aging in human skeletal muscle. Exp. Gerontol..

[64] Beregi E., Regius O. (1987). Comparative morphological study of age related mitochondrial changes of the lymphocytes and skeletal muscle cells. Acta Morphol. Hung..

[65] Greco M., Villani G., Mazzucchelli F., Bresolin N., Papa S., Attardi G. (2003). Marked aging-related decline in efficiency of oxidative phosphorylation in human skin fibroblasts. FASEB J. Off. Publ. Fed. Am. Soc. Exp. Biol..

[66] Papa S. (1996). Mitochondrial oxidative phosphorylation changes in the life span. Molecular aspects and physiopathological implications. Biochim. Biophys. Acta (BBA) Bioenerg..

[67] Short K., Bigelow M., Kahl J., Singh R., Coenen-Schimke J., Raghavakaimal S. (2005). Decline in skeletal muscle mitochondrial function with aging in humans. Proc. Natl. Acad. Sci. USA.

[68] Johnson M.L., Robinson M.M., Nair K.S. (2013). Skeletal muscle aging and the mitochondrion. Trends Endocrinol. Metab..

[69] Petersen K., Befroy D., Dufour S., Dziura J., Ariyan C., Rothman D. (2003). Mitochondrial dysfunction in the elderly: Possible role in insulin resistance. Science.

[70] Crane J., Devries M., Safdar A., Hamadeh M., Tarnopolsky M. (2010). The effect of aging onhuman skeletal muscle mitochondrial and intramyocellular lipid ultrastructure. J. Gerontol. A Biol. Sci. Med. Sci..

[71] Lanza I., Nair K.S. (2010). Mitochondrial function as a determinant of life span. Pflug. Arch. Eur. J. Physiol..

[72] Richter C., Park J.W., Ames B.N. (1988). Normal oxidative damage to mitochondrial and nuclear DNA is extensive. Proc. Natl. Acad. Sci. USA.

[73] Yakes F.M., Van Houten B. (1997). Mitochondrial DNA damage is more extensive and persists longer than nuclear DNA damage in human cells following oxidative stress. Proc. Natl. Acad. Sci. USA.

[74] Johnson F., Sinclair D., Guarente L. (1999). Molecular biology of aging. Cell.

[75] Aiken J., Bua E., Cao Z., Lopez M., Wanagat J., McKenzie D., McKiernan S. (2002). Mitochondrial DNA deletion mutations and sarcopenia. Ann. N. Y. Acad. Sci..

[76] Bua E., Johnson J., Herbst A., Delong B., McKenzie D., Salamat S., Aiken J.M. (2006). Mitochondrial DNA deletion mutations accumulate intracellularly to detrimental levels in aged human skeletal muscle fibers. Am. J. Hum. Genet..

[77] Corral-Debrinski M., Horton T., Lott M.T., Shoffner J.M., Flint Beal M., Wallace D.C. (1992). Mitochondrial DNA deletions in human brain: Regional variability and increase with advanced age. Nat. Genet..

[78] Katayama M., Tanaka M., Yamamoto H., Ohbayashi T., Nimura Y., Ozawa T. (1991). Deleted mitochondrial DNA in the skeletal muscle of aged individuals. Biochem. Int..

[79] Lee H.-C., Pang C.-Y., Hsu H.-S., Wei Y.-H. (1994). Differential accumulations of 4977 bp deletion in mitochondrial DNA of various tissues in human ageing. Biochim. Biophys. Acta (BBA) Mol. Basis Dis..

[80] Melov S., Shoffner J.M., Kaufman A., Wallace D.C. (1995). Marked increase in the number and variety of mitochondrial DNA rearrangements in aging human skeletal muscle. Nucleic Acids Res..

[81] Lee H.-C., Pang C.-Y., Hsu H.-S., Weia Y.-H. (1994). Ageing-associated tandem duplications in the D-loop of mitochondrial DNA of human muscle. FEBS Lett..

[82] Zhang C.F., Linnane A.W., Nagley P. (1993). Occurrence of a particular base substitution (3243 A to G) in mitochondrial DNA of tissues of ageing humans. Biochem. Biophys. Res. Commun..

[83] Cao Z., Wanagat J., McKiernan S.H., Aiken J.M. (2001). Mitochondrial DNA deletion mutations are concomitant with ragged red regions of individual, aged muscle fibers: Analysis by laser-capture microdissection. Nucleic Acids Res..

[84] Pak J.W., Herbst A., Bua E., Gokey N., McKenzie D., Aiken J.M. (2003). Mitochondrial DNA mutations as a fundamental mechanism in physiological declines associated with aging. Aging Cell.

[85] Wanagat J., Cao Z., Pathare P., Aiken J.M. (2001). Mitochondrial DNA deletion mutations colocalize with segmental electron transport system abnormalities, muscle fiber atrophy, fiber splitting, and oxidative damage in sarcopenia. FASEB J. Off. Publ. Fed. Am. Soc. Exp. Biol..

[86] Brierley E.J., Johnson M.A., Lightowlers R.N., James O.F., Turnbull D.M. (1998). Role of mitochondrial DNA mutations in human aging: Implications for the central nervous system and muscle. Ann. Neurol..

[87] Krishnan K.J., Reeve A.K., Samuels D.C., Chinnery P.F., Blackwood J.K., Taylor R.W., Wanrooij S., Spelbrink J.N., Lightowlers R.N., Turnbull D.M. (2008). What causes mitochondrial DNA deletions in human cells?. Nat. Genet..

[88] Chabi B., de Camaret B.M., Chevrollier A., Boisgard S., Stepien G. (2005). Random mtDNA deletions and functional consequence in aged human skeletal muscle. Biochem. Biophys. Res. Commun..

[89] Scatena R., Bottoni P., Giardina B. (2012). Advances in Mitochondrial Medicine.

[90] Alway S., Myers M., Mohamed J. (2014). Regulation of satellite cell function in sarcopenia. Front. Aging Neurosci..

[91] Dickinson J.M., Volpi E., Rasmussen B.B. (2013). Exercise and nutrition to target protein synthesis impairments in aging skeletal muscle. Exerc. Sport Sci. Rev..

[92] Breen L., Phillips S.M. (2011). Skeletal muscle protein anabolism in elderly: Intervention to counteract the “anabolic resistance” of ageing. Nutr. Metab..

[93] Kumar V., Selby A., Rankin D., Patel R., Atherton P., Hildebrandt W., Williams J., Smith K., Seynnes O., Hiscock N. (2009). Age-related differences in the dose–response relationship of muscle protein synthesis to resistance exercise in young and old men. J. Physiol..

[94] Churchward-Venne T.A., Breen L., Phillips S.M. (2013). Alterations in human muscle protein metabolism with aging: Protein and exercise as countermeasures to offset sarcopenia. BioFactors.

[95] Linnane A., Ozawa T., Marzuki S., Tanaka M. (1989). Mitochondrial DNA mutations as an important contributor to aging and degenerative diseases. Lancet.

[96] Fischer F., Hamann A., Osiewacz H.D. (2012). Mitochondrial quality control: An integrated network of pathways. Trends Biochem. Sci..

[97] Jensen M.B., Jasper H. (2014). Mitochondrial Proteostasis in the Control of Aging and Longevity. Cell Metab..

[98] Lim J.-A., Li L., Raben N. (2014). Pompe disease: From pathophysiology to therapy and back again. Front. Aging Neurosci..

[99] Rubinsztein D., Marino G., Kroemer G. (2011). Autophagy and aging. Cell.

[100] Höhn A., König J., Grune T. (2013). Protein oxidation in aging and the removal of oxidized proteins. J. Proteom..

[101] Rajawat Y.S., Hilioti Z., Bossis I. (2009). Aging: Central role for autophagy and the lysosomal degradative system. Ageing Res. Rev..

[102] Cuervo A.M., Bergamini E., Brunk U.T., Droge W., Ffrench M., Terman A. (2005). Autophagy and aging: The importance of maintaining “clean” cells. Autophagy.

[103] Zhang C., Cuervo A.M. (2008). Restoration of chaperone-mediated autophagy in aging liver improves cellular maintenance and hepatic function. Nat. Med..

[104] Gray D.A., Woulfe J. (2005). Lipofuscin and aging: A matter of toxic waste. Sci. Aging Knowl. Knowl. Environ..

[105] Melis J.P., Jonker M.J., Vijg J., Hoeijmakers J.H., Breit T.M., Van Steeg H. (2013). Aging on a different scale chronological versus pathology-related aging. Aging.

[106] Höhn A., Grune T. (2013). Lipofuscin: Formation, effects and role of macroautophagy. Redox Biol..

[107] Hütter E., Skovbro M., Lener B., Prats C., Rabøl R., Dela F., Jansen-Dürr P. (2007). Oxidative stress and mitochondrial impairment can be separated from lipofuscin accumulation in aged human skeletal muscle. Aging Cell.

[108] Terman A., Kurz T. (2013). Lysosomal iron, iron chelation, and cell death. Antioxid. Redox Signal..

[109] Mrschtik M., Ryan K.M. (2015). Lysosomal proteins in cell death and autophagy. FEBS J..

[110] Hornung V., Latz E. (2010). Critical functions of priming and lysosomal damage for NLRP3 activation. Eur. J. Immunol..

[111] Medzhitov R. (2008). Origin and physiological roles of inflammation. Nature.

[112] Álvarez-Rodríguez L., López-Hoyos M., Muñoz-Cacho P., Martínez-Taboada V.M. (2012). Aging is associated with circulating cytokine dysregulation. Cell. Immunol..

[113] Franceschi C., Bonafe M., Valensin S., Olivieri F., De Luca M., Ottaviani E., De Benedictis G. (2000). Inflamm-aging: An evolutionary perspective on immunosenescence. Ann. N. Y. Acad. Sci..

[114] Krabbe K.S., Pedersen M., Bruunsgaard H. (2004). Inflammatory mediators in the elderly. Exp. Gerontol..

[115] Pinti M., Cevenini E., Nasi M., De Biasi S., Salvioli S., Monti D., Benatti S., Gibellini L., Cotichini R., Stazi M.A. (2014). Circulating mitochondrial DNA increases with age and is a familiar trait: Implications for “inflamm-aging”. Eur. J. Immunol..

[116] Singh T., Newman A.B. (2010). Inflammatory markers in population studies of aging. Ageing Res. Rev..

[117] Jo E., Lee S.-R., Park B.-S., Kim J.-S. (2012). Potential mechanisms underlying the role of chronic inflammation in age-related muscle wasting. Aging Clin. Exp. Res..

[118] Simpson R.J., Lowder T.W., Spielmann G., Bigley A.B., LaVoy E.C., Kunz H. (2012). Exercise and the aging immune system. Ageing Res. Rev..

[119] Martinon F., Burns K., Tschopp J. (2002). The inflammasome: A molecular platform triggering activation of inflammatory caspases and processing of pro IL-β. Mol. Cell.

[120] Yazdi A., Drexler S., Tschopp J. (2010). The role of the inflammasome in nonmyeloid cells. J. Clin. Immunol..

[121] Martinon F., Mayor A., Tschopp J. (2009). The inflammasomes: Guardians of the body. Annu. Rev. Immunol..

[122] Tschopp J. (2011). Mitochondria: Sovereign of inflammation?. Eur. J. Immunol..

[123] Zhou R., Yazdi A.S., Menu P., Tschopp J. (2011). A role for mitochondria in NLRP3 inflammasome activation. Nature.

[124] Gurung P., Lukens J.R., Kanneganti T.-D. (2014). Mitochondria: Diversity in the regulation of the NLRP3 inflammasome. Trends Mol. Med..

[125] Shimada K., Crother T.R., Karlin J., Dagvadorj J., Chiba N., Chen S., Ramanujan V.K., Wolf A.J., Vergnes L., Ojcius D.M. (2012). Oxidized mitochondrial DNA activates the NLRP3 inflammasome during apoptosis. Immunity.

[126] Goto S., Naito H., Kaneko T., Chung H.Y., Radak Z. (2007). Hormetic effects of regular exercise in aging: Correlation with oxidative stress. Appl. Physiol. Nutr. Metab..

[127] Zealley B., De Grey A.D. (2013). Strategies for engineered negligible senescence. Gerontology.

[128] Piantadosi C.A., Suliman H.B. (2012). Redox regulation of mitochondrial biogenesis. Free Radic. Biol. Med..

[129] He C., Bassik M.C., Moresi V., Sun K., Wei Y., Zou Z., An Z., Loh J., Fisher J., Sun Q. (2012). Exercise-induced BCL2-regulated autophagy is required for muscle glucose homeostasis. Nature.

[130] He C., Sumpter J.R., Levine B. (2012). Exercise induces autophagy in peripheral tissues and in the brain. Autophagy.

[131] Pagano A.F., Py G., Bernardi H., Candau R.B., Sanchez A.M.J. (2014). Autophagy and protein turnover signaling in slow-twitch muscle during exercise. Med. Sci. Sports Exerc..

[132] Sanchez A.M., Bernardi H., Py G., Candau R. (2014). Autophagy is essential to support skeletal muscle plasticity in response to endurance exercise. Am. J. Physiol. Regul. Integr. Comp. Physiol..

[133] Powers S.K., Nelson W.B., Hudson M.B. (2011). Exercise-induced oxidative stress in humans: Cause and consequences. Free Radic. Biol. Med..

[134] Ji L.L., Gomez-Cabrera M.-C., Vina J. (2007). Role of nuclear factor kB and mitogen-activated protein kinase signaling in exercise-induced antioxidant enzyme adaptation. Appl. Physiol. Nutr. Metab..

[135] Gounder S.S., Kannan S., Devadoss D., Miller C.J., Whitehead K.S., Odelberg S.J., Firpo M.A., Paine R., Hoidal J.R., Abel E.D. (2012). Impaired transcriptional activity of Nrf2 in age-related myocardial oxidative stress is reversible by moderate exercise training. PLoS ONE.

[136] MacNeil L.G., Safdar A., Baker S.K., Melov S., Tarnopolsky M.A. (2011). Eccentric exercise affects Nrf2-mediated oxidative stress response in skeletal muscle by increasing nuclear Nrf2 content. Med. Sci. Sports Exerc..

[137] Gleeson M., Bishop N.C., Stensel D.J., Lindley M.R., Mastana S.S., Nimmo M.A. (2011). The anti-inflammatory effects of exercise: Mechanisms and implications for the prevention and treatment of disease. Nat. Rev. Immunol..

[138] Petersen A., Pedersen B. (2005). The anti-inflammatory effect of exercise. J. Appl. Physiol..

[139] Pedersen B., Febbraio M. (2008). Muscle as an endocrine organ: Focus on muscle-derived interleukin-6. Physiol. Rev..

[140] Bell K.E., Seguin C., Parise G., Baker S.K., Phillips S.M. (2015). Day-to-day changes in muscle protein synthesis in recovery from resistance, aerobic, and high-intensity interval exercise in older men. J. Gerontol. Ser. A.

[141] Tapia P.C. (2006). Sublethal mitochondrial stress with an attendant stoichiometric augmentation of reactive oxygen species may precipitate many of the beneficial alterations in cellular physiology produced by caloric restriction, intermittent fasting, exercise and dietary phytonutrients: Mitohormesis for health and vitality. Med. Hypotheses.

[142] Menshikova E.V., Ritov V.B., Fairfull L., Ferrell R.E., Kelley D.E., Goodpaster B.H. (2006). Effects of exercise on mitochondrial content and function in aging human skeletal muscle. J. Gerontol. Ser. A Biol. Sci. Med Sci..

[143] Short K., Vittone J., Bigelow M. (2003). Impact of aerobic exercise training on age-related changes in insulin sensitivity and muscle oxidative capacity. Diabetes Care.

[144] Broskey N.T., Greggio C., Boss A., Boutant M., Dwyer A., Schlueter L., Hans D., Gremion G., Kreis R., Boesch C. (2015). Skeletal muscle mitochondria in the elderly: Effects of physical fitness and exercise training. J. Clin. Endocrinol. Metab..

[145] Conley K., Jubrias S., Cress M., Esselman P. (2013). Elevated energy coupling and aerobic capacity improves exercise performance in endurance-trained elderly subjects. Exp. Physiol..

[146] Jubrias S.A., Esselman P.C., Price L.B., Cress M.E., Conley K.E. (2001). Large energetic adaptations of elderly muscle to resistance and endurance training. J. Appl. Physiol..

[147] Jacobs R.A., Lundby C. (2013). Mitochondria express enhanced quality as well as quantity in association with aerobic fitness across recreationally active individuals up to elite athletes. J. Appl. Physiol..

[148] Parise G., Brose A.N., Tarnopolsky M.A. (2005). Resistance exercise training decreases oxidative damage to DNA and increases cytochrome oxidase activity in older adults. Exp. Gerontol..

[149] Parise G., Phillips S.M., Kaczor J.J., Tarnopolsky M.A. (2005). Antioxidant enzyme activity is up-regulated after unilateral resistance exercise training in older adults. Free Radic. Biol. Med..

[150] Ji L.L., Zhang Y. (2014). Antioxidant and anti-inflammatory effects of exercise: Role of redox signaling. Free Radic. Res..

[151] Melov S., Tarnopolsky M.A., Beckman K., Felkey K., Hubbard A. (2007). Resistance exercise reverses aging in human skeletal muscle. PLoS ONE.

[152] Tarnopolsky M., Zimmer A., Paikin J., Safdar A., Aboud A., Pearce E., Roy B., Doherty T. (2007). Creatine monohydrate and conjugated linoleic acid improve strength and body composition following resistance exercise in older adults. PLoS ONE.

[153] Tarnopolsky M.A. (2009). Mitochondrial DNA shifting in older adults following resistance exercise training. Appl. Physiol. Nutr. Metab..

[154] Pesta D., Hoppel F., Macek C., Messner H., Faulhaber M., Kobel C., Parson W., Burtscher M., Schocke M., Gnaiger E. (2011). Similar qualitative and quantitative changes of mitochondrial respiration following strength and endurance training in normoxia and hypoxia in sedentary humans. Am. J. Physiol. Regul. Integr. Comp. Physiol..

[155] Porter C., Reidy P., Bhattarai N., Sidossis L., Rasmussen B. (2014). Resistance exercise training alters mitochondrial function in human skeletal muscle. Med. Sci. Sports Exerc..

[156] Taivassalo T., Fu K., Johns T., Arnold D., Karpati G., Shoubridge E.A. (1999). Gene shifting: A novel therapy for mitochondrial myopathy. Hum. Mol. Genet..

[157] Murphy J.L., Blakely E.L., Schaefer A.M., He L., Wyrick P., Haller R.G., Taylor R.W., Turnbull D.M., Taivassalo T. (2008). Resistance training in patients with single, large-scale deletions of mitochondrial DNA. Brain.

[158] Ji L.L. (2001). Exercise at old age: Does it increase or alleviate oxidative stress?. Ann. N. Y. Acad. Sci..

[159] Izzotti A. (2011). Genomic biomarkers and clinical outcomes of physical activity. Ann. N. Y. Acad. Sci..

[160] Couppe C., Svensson R.B., Grosset J.-F., Kovanen V., Nielsen R., Olsen M., Larsen J., Praet S.E., Skovgaard D., Hansen M. (2013). Life-long endurance running is associated with reduced glycation and mechanical stress in connective tissue. Age.

[161] Head D., Bugg J.M., Goate A.M., Fagan A.M., Mintun M.A., Benzinger T., Holtzman D.M., Morris J.C. (2012). Exercise engagement as a moderator of the effects of apoe genotype on amyloid deposition. Arch. Neurol..

[162] Tam B.T., Siu P.M. (2014). Autophagic cellular responses to physical exercise in skeletal muscle. Sports Med..

[163] Erlich A.T., Brownlee D.M., Beyfuss K., Hood D.A. (2018). Exercise induces TFEB expression and activity in skeletal muscle in a PGC-1α-dependent manner. Am. J. Physiol. Cell Physiol..

[164] Kim Y., Hood D.A. (2017). Regulation of the autophagy system during chronic contractile activity-induced muscle adaptations. Physiol. Rep..

[165] Lira V.A., Okutsu M., Zhang M., Greene N.P., Laker R.C., Breen D.S., Hoehn K.L., Yan Z. (2013). Autophagy is required for exercise training-induced skeletal muscle adaptation and improvement of physical performance. FASEB J. Off. Publ. Fed. Am. Soc. Exp. Biol..

[166] Pedersen B.K., Ostrowski T., Rohde K., Bruunsgaard H. (1998). The cytokine response to strenuous exercise. Can. J. Physiol. Pharm..

[167] Proske U., Morgan D.L. (2001). Muscle damage from eccentric exercise: Mechanism, mechanical signs, adaptation and clinical applications. J. Physiol..

[168] Greiwe J.S., Cheng B.O., Rubin D.C., Yarasheski K.E., Semenkovich C.F. (2001). Resistance exercise decreases skeletal muscle tumor necrosis factor alpha in frail elderly humans. FASEB J. Off. Publ. Fed. Am. Soc. Exp. Biol..

[169] McFarlin B.K., Flynn M.G., Phillips M.D., Stewart L.K., Timmerman K.L. (2005). Chronic resistance exercise training improves natural killer cell activity in older women. J. Gerontol. A Biol. Sci. Med. Sci..

